# DFT insights into humic acid coordination of Cd(II) Cu(II) and Pb(II)

**DOI:** 10.1038/s41598-025-34197-8

**Published:** 2026-01-21

**Authors:** Hanan Elhaes, Medhat A. Ibrahim

**Affiliations:** 1 Physics Department, Faculty of Women for Arts, Science and Education, Ain Shams University, Cairo, 11757 Egypt; 2 Spectroscopy Department, National Research Centre, 33 El-Bohouth St, Dokki, 12622 Giza Egypt; 3 Molecular Modeling and Spectroscopy Laboratory, Centre of Excellence for Advanced Science, National Research Centre, 33 El-Bohouth St, Dokki, 12622 Giza Egypt

**Keywords:** Cd, Cu, Pb, DFT, MP2 and humic acid HA, Chemistry, Environmental sciences

## Abstract

This study used Density Functional Theory (DFT) at the B3LYP/6-31G(d, p) level to develop a model for humic acid (HA), characterizing it as an organic molecule with functional groups featuring hydrogen bonding. The calculated Infrared (IR) spectrum of the HA model matched experimental results from Fourier-Transform Infrared (FTIR) spectroscopy. Analysis using Molecular Electrostatic Potential (MESP) maps and Highest Occupied/Lowest Unoccupied Molecular Orbitals (HOMO/LUMO) suggested that HA could be simplified to a reactive R-COOH (carboxylic acid) model. A computational comparison between B3LYP/6-31G(d, p), MP2, and the semi-empirical PM6 method found that PM6 was suitable for studying the R-COOH model, offering a balance of accuracy and computational efficiency. The coordinating ability of the divalent metals Cd, Cu, and Pb was investigated, showing they can coordinate with two R-COOH units. Cu and Pb were found to be more reactive than the coordinated Cd. Further simulations, where each metal was hydrated with four water molecules, revealed that the hydrated coordinated metals were more reactive than their non-hydrated counterparts. A general model for metal/HA coordination was explored by interacting hydrated Cu with two full HA units. It was concluded that HA is a useful agent for coordinating divalent metals in aquatic environments. However, the Quantum Theory of Atoms in Molecules (QTAIM) analysis indicated that this coordination process might negatively affect the HA’s stability and environmental degradation.

## Introduction

Humic substances are natural complex mixtures of organic molecules which are formed as a result of the decomposition process for living matter such as plants and animals. They could be classified as part of organic matter in soils as well as aquatic environments^1,2^. Generally, they play crucial roles in soil quality, nutrient cycling, and carbon sequestration^3^. Based on the solubility of humic substances under different pH conditions, it could be classified into three common fractions namely: humic acids, fulvic acids, and humin^4^. Humic acid HA is organic macromolecule which is classified among the most essential parts of humic substances, which has many oxygen rich functional groups^5,6^. HA is accumulated the remains of both plant and/or animal throughout bio-decomposition and transformation, which leads to complex structures. This enable HA for several applications in many areas like agriculture, forestry, animal husbandry, environmental protection, as well as the petroleum industry^7^. HA shows potential application as fertilizer in one hand and could remediate heavy metals on the other^8^. HA is responsible for improving the soil’s physical and chemical properties which in turn leads to an enhancement of both plant growth and development^8,9^. Rather than the mentioned applications HA shows also potential for application in biomedical fields^10^. One of the important role of HA is its role in precipitating heavy metals which reduces the mobility of these traces in soil then control their uptake by plants^11^. This role of HA could increase soil nutrients availability, throughout micronutrients by chelating and co-transporting them to plants^12,13^. It was reported that, the HA controls the soils system as a part of sustainable approach for controlling and adapting the climate change^14^.

From the above consideration one can conclude that, HA could be used for managing heavy and trace metals in the environment especially in soil. This process could be conducted by the rich functional groups in HA which could bind and stabilize these metals, this process could reduce both the mobility and bioavailability of trace metals. This process could be in general responsible for controlling the fate and transport heavy metal in the environment^15–17^.

Understanding the mode and mechanism of action for HA is important to understand its fate and functions. Computational methods especially those based on quantum mechanical calculations are very important to study different molecular systems^18^. Physical parameters such as total dipole moment, HOMO/LUMO energy and electrostatic potential could be good indicators for the reactivity of a given molecular system^19–21^. Some researchers pointed out that, the stability of the studied compounds could be also derived from some parameters obtained by molecular modeling^22,23^. Periodic DFT + U calculations to investigate the remediation of uranium contamination by reducing highly mobile U(VI) to the less soluble U(IV) at mineral surfaces. The study offers crucial insights into the mechanisms of U(VI) reduction and the dual role of humic acid (HA) in this process, highlighting both competitive and synergistic effects^24^.

The aim of the study is to use quantum chemical calculations to elucidate the molecular-level details of how three divalent heavy metals Cd(II), Cu(II), and Pb(II) coordinate with the reactive carboxylic functional groups of humic acid (HA), focusing on comparative reactivity and the influence of the aquatic environment. So that, a model of R-COOH is proposed to simulate HA whereas each metal is interacting through hydrogen bond of two units of HA. The HOMO/LUMO orbitals and MESP will be conducted to follow up them for HA as well as HA interacted with Cd, Cu and Pb respectively. Different level of theory such as B3LYP/6-31G(d, p), MP2 and PM6 were utilized while general model of HA was calculated with B3LYP/6-31G(d, p) level of theory. The role of the aqueous environment is elucidated by comparing the coordination parameters of both non-hydrated (M(R-COOH)_2_​) and hydrated (M(R-COOH)_2_​⋅(H_2_​O)_4_​) complexes.

## Molecular modeling calculations

### Building model molecules

**Fig. 1:**
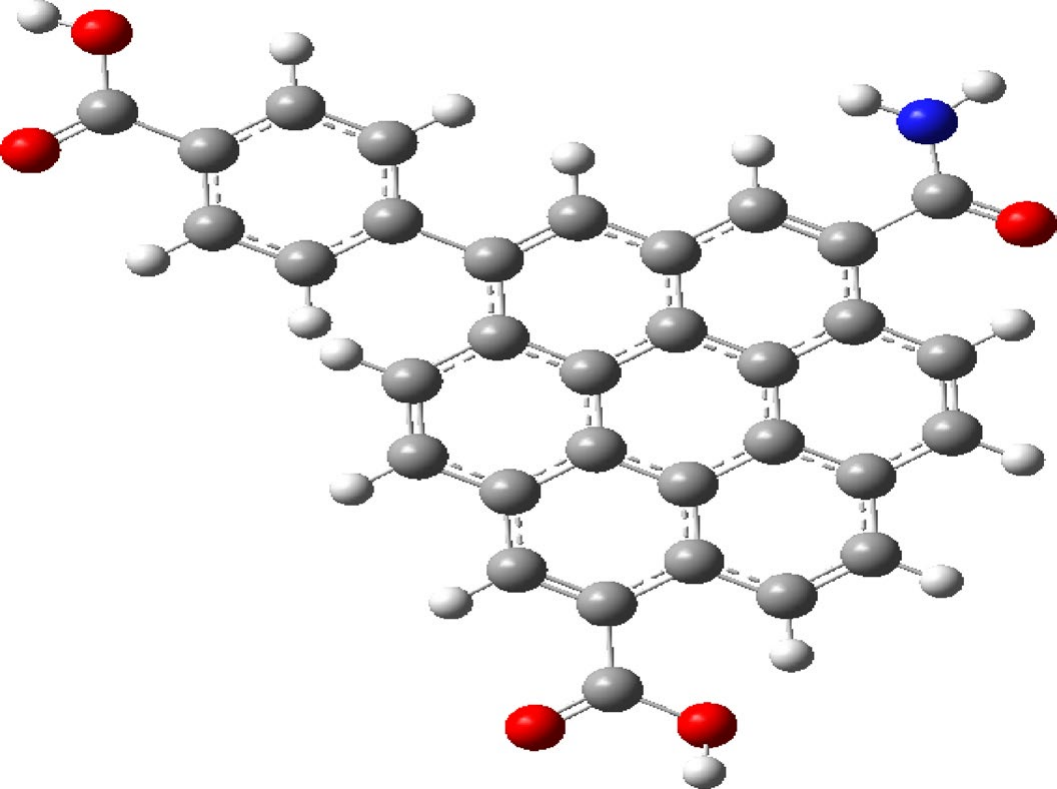
Model structure for humic acid HA.

**Fig. 2:**
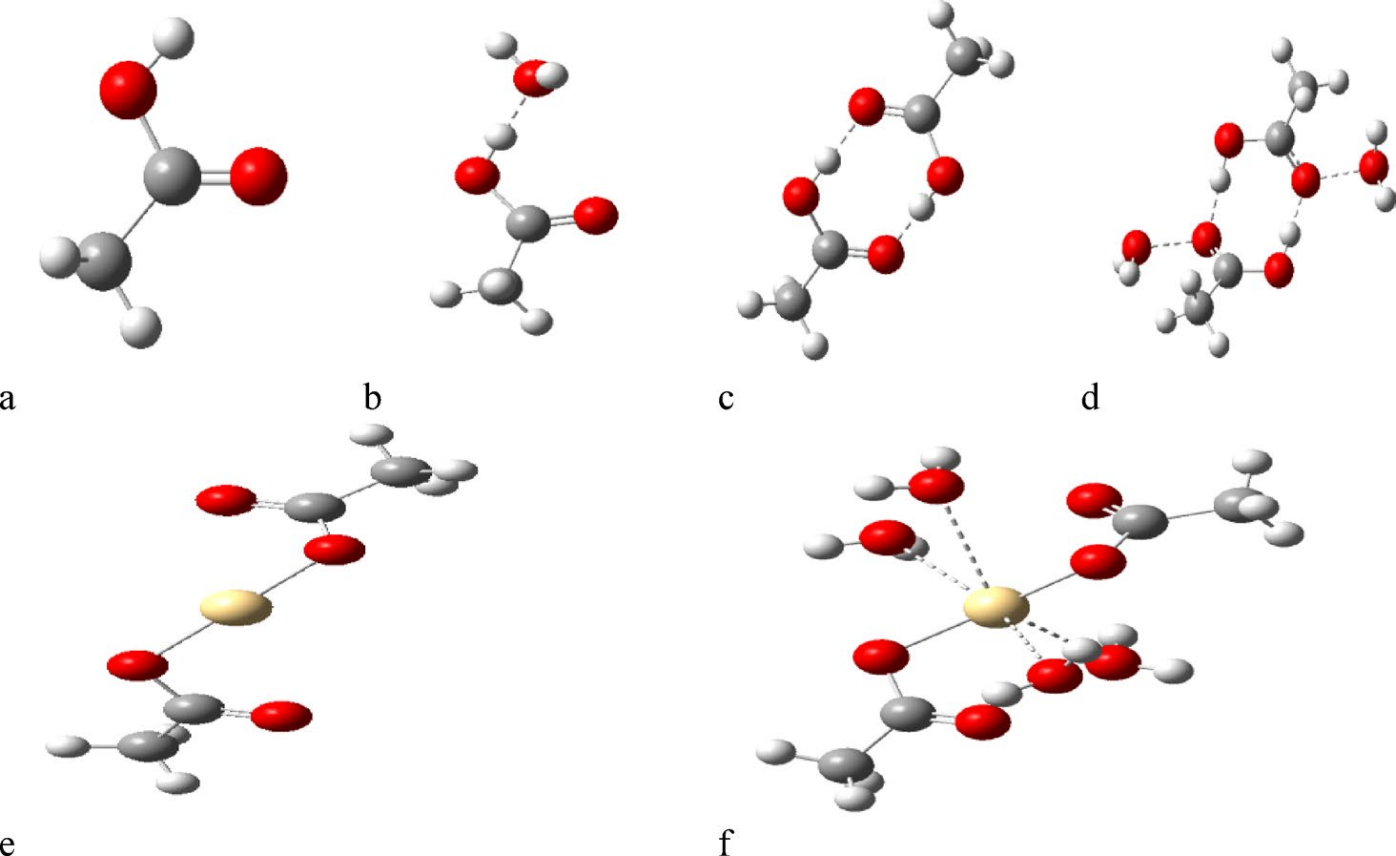
Model structure for (**a**) acetic acid, (**b**) acetic acid with one water molecule, (**c**) acetic acid dimer and (**d**) acetic acid dimer with two water molecules, (**e**) Cd interacted with two acetic acid units throughout H of COOH group and (**f**) Cd hydrated with 4 water molecules interacted with two acetic acid units throughout H of COOH group. The same is repeated in case of Cu and Pb.

As indicated in Fig. 1, a model for humic acid is indicated, the model is a class of hydrocarbon chain with both aliphatic and aromatic chain coupled with functional groups. To describe the process of coordination of divalent metals such as Cd, Cu and Pb the coordination is supposed to take place with the hydrogen bonding of COOH group. Accordingly, the model for humic acid is further simplified to be acetic acid as indicated in Fig. 2a, with possible hydration with one water molecule as shown in Fig. 2b. then acetic acid is dimerized as indicated in Fig. 2c and finally acetic acid dimer with two water molecules as shown in Fig. 2d.

To simulate the coordination of the studied metals with the humic acid two assumption are tested as indicated in Fig. 2e and -f. Each metal Cd, Cu and Pb is supposed to interact with two hydrogen bonds of COOH forming the model indicated in Fig. 2e. the same model is supposed to interacting with 4 water molecules around the divalent metal as indicated in Fig. 2f. All the studied models were built with the help of GaussView 5.0 software^25^.

These assumptions for possible coordination of divalent metals by humic acid is then tested with density functional theory DFT, the details of computation will be described as in the following section.

### Computational details

The model molecule for humic acid was subjected to optimization using G09 program^26^ which implemented on a personal computer at the Molecular Modeling and Spectroscopy Laboratory, Centre of Excellence for Advanced Science, National Research Centre, Egypt. The humic acid molecule is optimized with B3LYP^27–29^ together with 6-31G(d, P) basis set, the IR frequencies were calculated at the same level of theory. Acetic acid was subjected to optimization with B3LYP/6-31G(d, p), MP2 and PM6 level. Then, the models for possible coordination between Cd, Cu and Pb with acetic acid were optimized with PM6. Some important parameters were calculated at the same level of theory such as total dipole moment, HOMO/LUMO band gap energy, molecular electrostatic potential MESP and density of states DOS. Based on HOMO and LUMO energy some important descriptors called global reactivity were calculated such as ionization potential (I), electronic affinity (A), chemical potential (µ), chemical hardness (η), absolute chemical softness (S) and electrophilicity index (ω). These descriptors were computed with the following Equations^30,31^:

**Table:** 

I = -E_HOMO_
A = E_LUMO_
µ = -(I + A)/2
η = (I-A)/2
S = 1/η
ω = µ^2^/2η

The quantum theory of atoms in molecules QTAIM calculations were conducted with the help of Avogadro software^32^. The QTAIM was conducted as a test for the stability of the studied model structures.

## Results and discussions

**Fig. 3:**
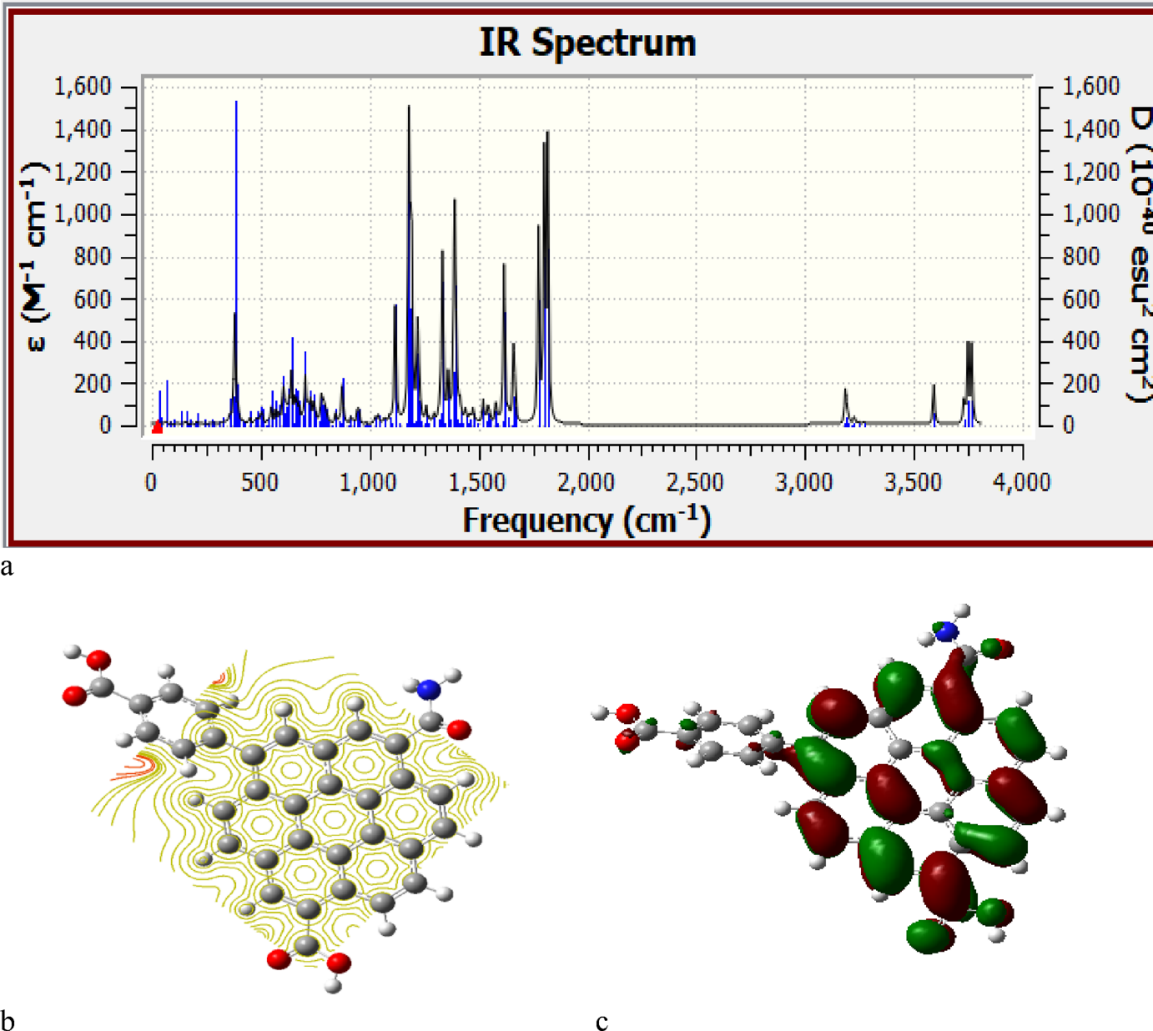
Calculated (**a**) IR frequencies for humic acid model which calculated at B3LYP/6-31G(d, p), (**b**) MESP map and (**c**)HOMO/LUMO orbitals.

Researchers can obtain HA from environmental samples, which vary from one another due to their sources and environmental conditions^33,34^. Thus, two samples taken from distinct locations can be reported, highlighting the primary functional groups as indicators for the potential structure of HA. According to the provided FTIR data^33,34^, the spectrum of HA can be categorized as shown in Table 1. The CH showed several bands for deformation vibrations at 472, 800, 993, 1158, 2800, and 2900 cm^− 1^. A band of N-H bending appears at 618 cm^− 1^. The Methyl symmetric bending vibration is found at 1380 cm-1. The peak at 1640 cm-1 is attributed to C = C stretching vibration, while the C = O of the COOH group is observed at 1721 cm^− 1^. The OH band is found between approximately 3300 and 3400 cm^− 1^. Using these spectral characteristics, the HA is formed as shown in Fig. 1. Figure 1 illustrated the model structure analyzed for HA, which is succinctly characterized as an organic framework modified with oxygen-rich functional groups as depicted in the figure. The determined IR for the examined humic acid model is shown in Fig. 3a. The calculated IR spectrum displays no negative frequencies, indicating the validity of the optimized structure obtained via B3LYP/6-31G(d, P).

Table 1 showed a comparison between FTIR and calculated IR at B3LYP/6-31G(d, P). Results showed that the modeled data aligns with the experimental FTIR findings. It has been mentioned that metals in the environment may coordinate with HA due to the hydrogen bonding of the COOH group.

**Table 1:** Calculated IR frequencies for HA which calculated at B3LYP/6-31G(d, p), in comparison with FTIR spectrum of HA.

Calculated IR (cm^− 1^)		FTIR (cm^− 1^)	Band assignment
Unscaled	Scaled		
497.9	482.0	472	CH deformation vibration
656.0	635.2	618	N-H bending
873.8	845.7	800	CH deformation vibration
1118.6	1082.7	993	CH vibration
1181.3 1195.6	1143.3 1157.2	1158	CH vibration
1398.5 1416.0	1353.6 1370.5	1380	Methyl symmetric bending vibration
1668.4	1614.9	1640	C = C stretching vibration
1776.3 1818.7	1719.3 1760.3	1721	C = O
3186.5	3084.2	2800 2900	CH vibration
3749.7 3766.9	3629.3 3646.0	3300 3400	OH

The frequency scale factor is 0.9679^35^.

Table 2 displayed the global reactivity descriptors for humic acid, calculated using the B3LYP/6-31G(d, p) method, serving as the HA model molecule. The calculated descriptors suggested that the model is reactive, which may facilitate the coordination of metals due to the distinct hydrogen bonding present in functional groups like the COOH group. This result is supported by the MESP map shown in Fig. 3b, which demonstrates that the COOH is the reactive site, while HA can interact in the environment through the hydrogen of COOH. The MESP maps illustrate the potential interaction and/or molecular reactivity. The MESP color patterns indicate areas of electron abundance (nucleophilic) and electron scarcity (electrophilic). The color scheme could be arranged so that red indicates negative, blue signifies positive, and green represents neutral^36,37^. These maps merely illustrate and/or forecast how a particular molecule engages with its neighboring molecules. The HA displays a MESP outline represented by a contour with a yellow circle, signifying that HA is neutral.

**Table 2:** B3LYP/6-31G(d, p) calculated global reactivity descriptors for humic acid HA model molecule.

Structure	I (eV)	A (eV)	µ (eV)	η (eV)	S (eV^− 1^)	ω (eV)
HA	5.726409	2.082776	3.904592	1.82182	0.5489027	4.1842418

HOMO and LUMO refer to the highest occupied molecular orbitals and the lowest unfilled molecular orbital. HOMO/LUMO serves as key indicators for the reactivity, stability, and electronic characteristics of molecules^38^.

The HOMO/LUMO orbitals displayed in Fig. 3c demonstrate that the electrons in the orbitals surrounding the aliphatic COOH are more abundant than those around the aromatic COOH, suggesting a higher reactivity for aliphatic COOH in relation to aromatic COOH.

### Optimization of the computational method

A model molecule that explains specific phenomena can be simplified by incorporating a structure with the required functional group. This consequently accelerates the computational process. Thus, a model of HA might be useful for characterizing the nature of coordination instead of its duration. If HA is organizing metals via hydrogen bonding, an organic structure with a COOH group might be problematic in this scenario. The HA model will be represented as R-COOH as illustrated in Fig. 2a; this model could interact with one water molecule as shown in Fig. 2b. It can also form a dimer, as depicted in Fig. 2c, and then could dimerize and interact with two water molecules as indicated in Fig. 2d.

The simplified HA model denoted as R-COOH undergoes three calculations using three distinct computational techniques: DFT: B3LYP/6-31G(d, p), followed by MP2, and a semiempirical approach at PM6.

Table 3 shows the computed bond distances and bond angles for the acetic acid model molecule examined using B3LYP/6-31G(d, p), MP2, and PM6. The geometrical parameters shown in Table 3 demonstrate that the results are comparable for this small system. PM6 offers the quickest computation time. Based on the findings in Table 2, it was suggested to use PM6 to investigate the potential coordination between HA and specific divalent metals like Cd, Cu, and Pb. This will save computation time. Consequently, the PM6 will serve as the model for investigating the function of HA in the possible coordination of the chosen divalent metals, both with and without hydration.

**Table 3:** Calculated bond distances and bond angle for the studied acetic acid model molecule calculated at B3LYP/6-31G(d, p), MP2 and PM6.

Model	Bond distance of COOH group (Å)			Bond angle (⁰)
	L_C=O_	L_C−O_	L_O−H_	OCO
B3LYP/6-31G(d, p)	1.21021	1.35788	0.97214	122.47111
MP2	1.21742	1.36174	0.97103	122.67837
PM6	1.20746	1.37996	0.99355	119.53426

### Role of HA in metal coordination

Divalent metals like Cd, Cu, and Pb can coordinate with HA as illustrated in Fig. 2e, where each metal interacts with two hydrogen bonds, each associated with a single HA molecule. Another coordination is shown in Fig. 2f, which resembles Fig. 2e, but each metal is surrounded by four water molecules.

**Fig. 4:**
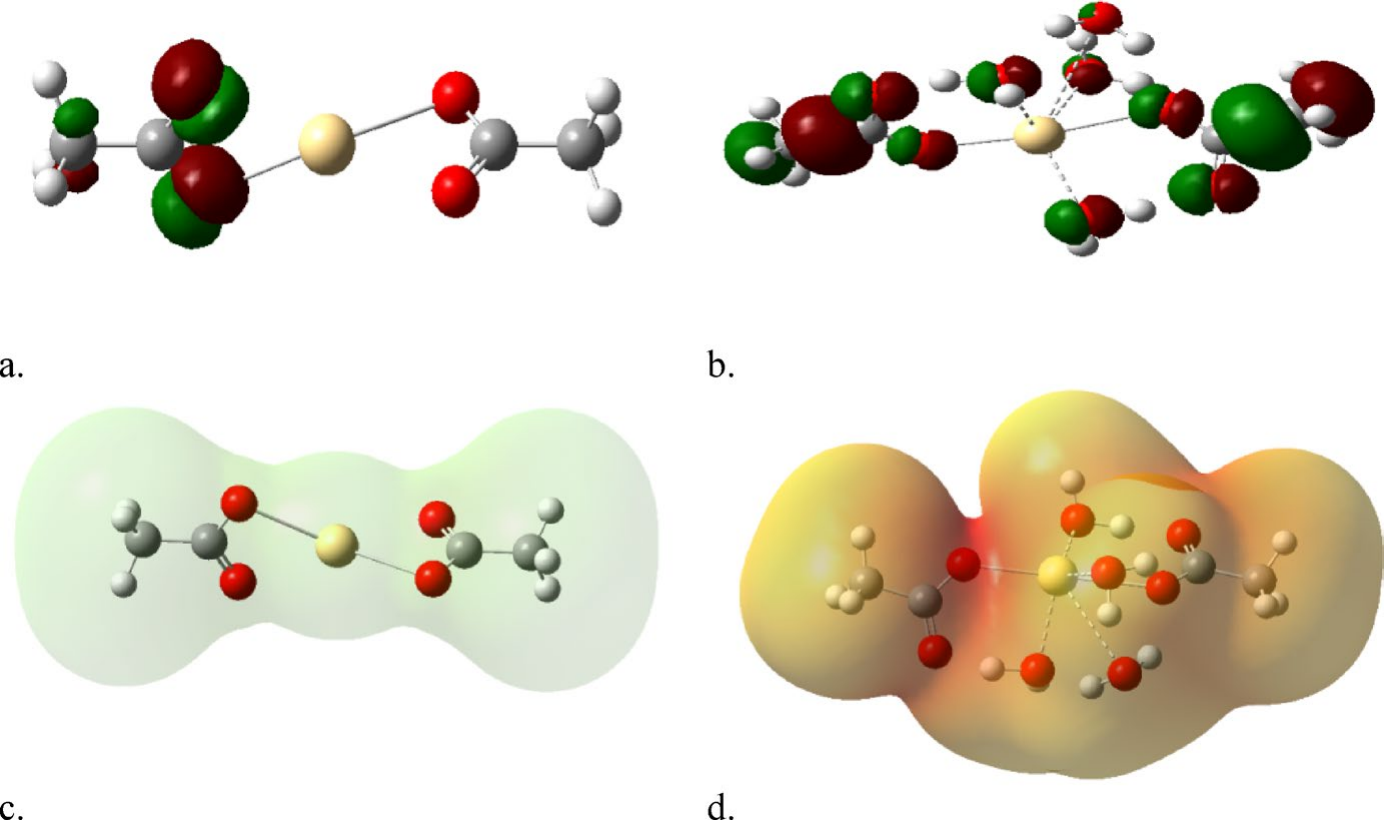
Calculated physical parameters for Cd coordinated with acetic acid without and with 4 water molecules whereas, (**a**), (**b**), HOMO/LUMO and (**c**), (**d**) MESP.

Figure 4 displays the calculated physical parameters for Cd coordinated with acetic acid both with and without 4 water molecules, while a, b, HOMO/LUMO, and c, d MESP were included. In Fig. 4a, the orbitals are positioned around the carboxyl group of the left HA model compound. When hydrated with four water molecules, the orbitals of both HA molecules are disrupted among the COOH. This indicates that hydration enhances the reactivity of HA coordination with Cd. The same is considered for mapped MESP in Fig. 4c and d, where the hydrated Cd displays a reactive surface of Cd coordinated with HA in contrast to non-hydrated Cd.

**Fig. 5:**
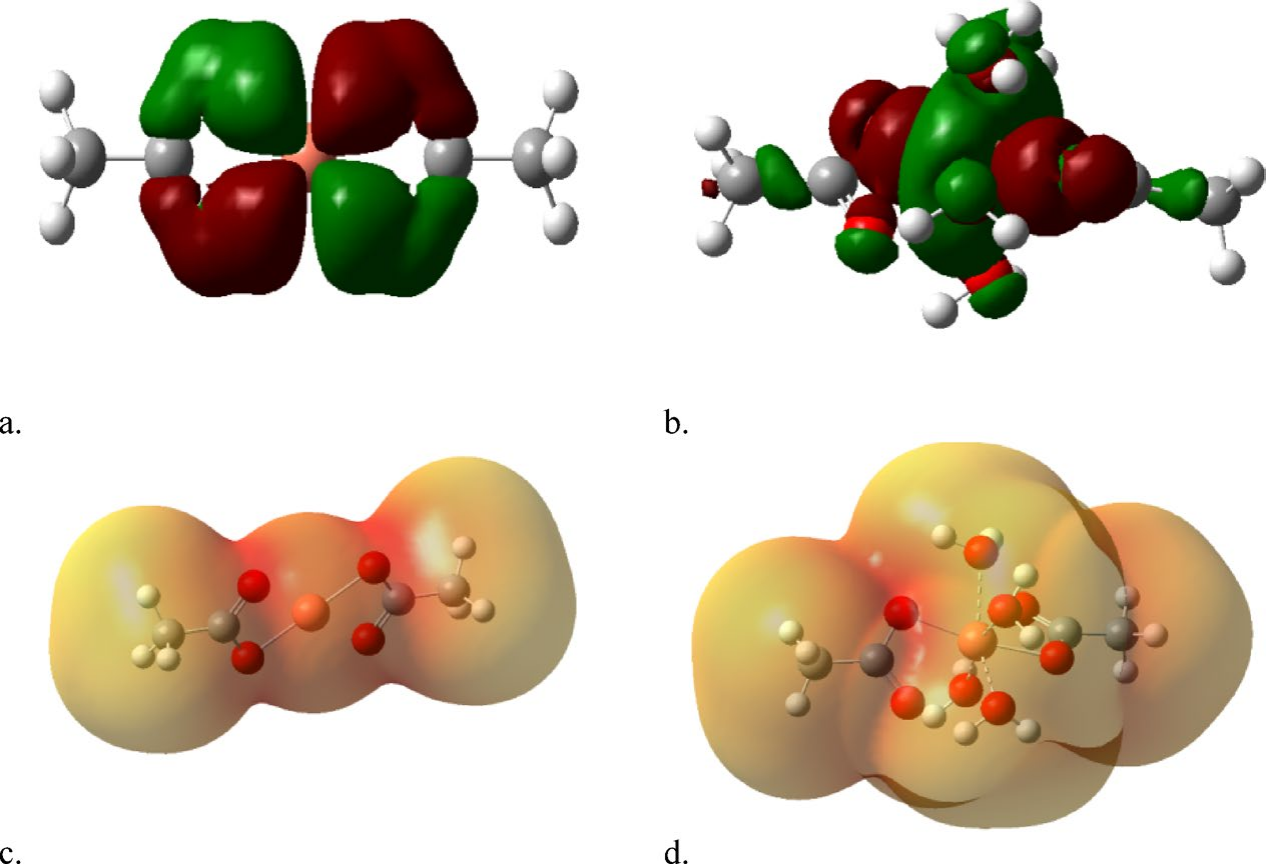
Calculated physical parameters for Cu coordinated with acetic acid without and with 4 water molecules whereas, (**a**), (**b**), HOMO/LUMO and (**c**), (**d**) MESP.

Figure 5a, b showed the calculated HOMO/LUMO for Cu coordinated with HA both without and with 4 water molecules. Results showed an uniform distribution of electrons on the carboxyl groups of the two HA model compounds, and the MESP maps in Fig. 5c, d illustrate the surface reactivity in both non-hydrated Cu (Fig. 5c) and hydrated Cu (Fig. 5d).

**Fig. 6:**
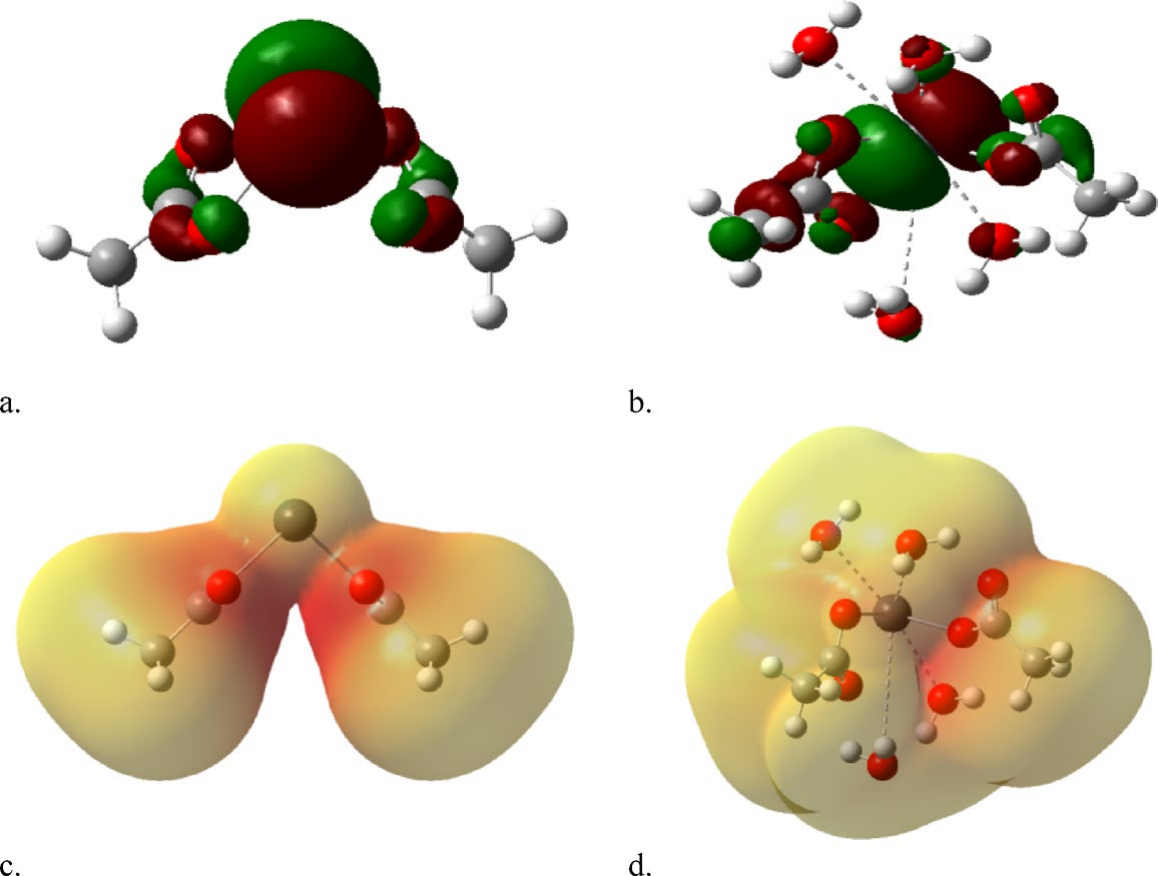
Calculated physical parameters for Pb coordinated with acetic acid without and with 4 water molecules whereas, (**a**), (**b**), HOMO/LUMO and (**c**), (**d**) MESP.

Figure 6 displayed results for Pb coordinated with HA both without and with 4 water molecules, while Fig. 6a, b illustrates HOMO/LUMO and Fig. 6c, d shows MESP. The coordination of Pb aligns well with the results observed for Cu coordinated with HA, as previously shown in Fig. 5.

The analysis of MESP and HOMO/LUMO maps for all three metals leads to two main correlated conclusions. The first one, for all metals (Cd, Cu, Pb), the presence of four coordinated water molecules enhances the metal-ligand interaction. For Cd, this is clearly demonstrated by the HOMO/LUMO orbitals shifting from a localized state to a disrupted, more uniform distribution, which MESP confirms by displaying a more reactive surface.

The second one, the MESP and HOMO/LUMO findings consistently indicated that the divalent metals remain electronically active even after coordination with the HA model. This suggests that the resulting HA-metal complexes are not inert, maintaining a potential for further interaction or reaction in the environment.

### General model for HA coordinated with heavy metals

**Fig. 7:**
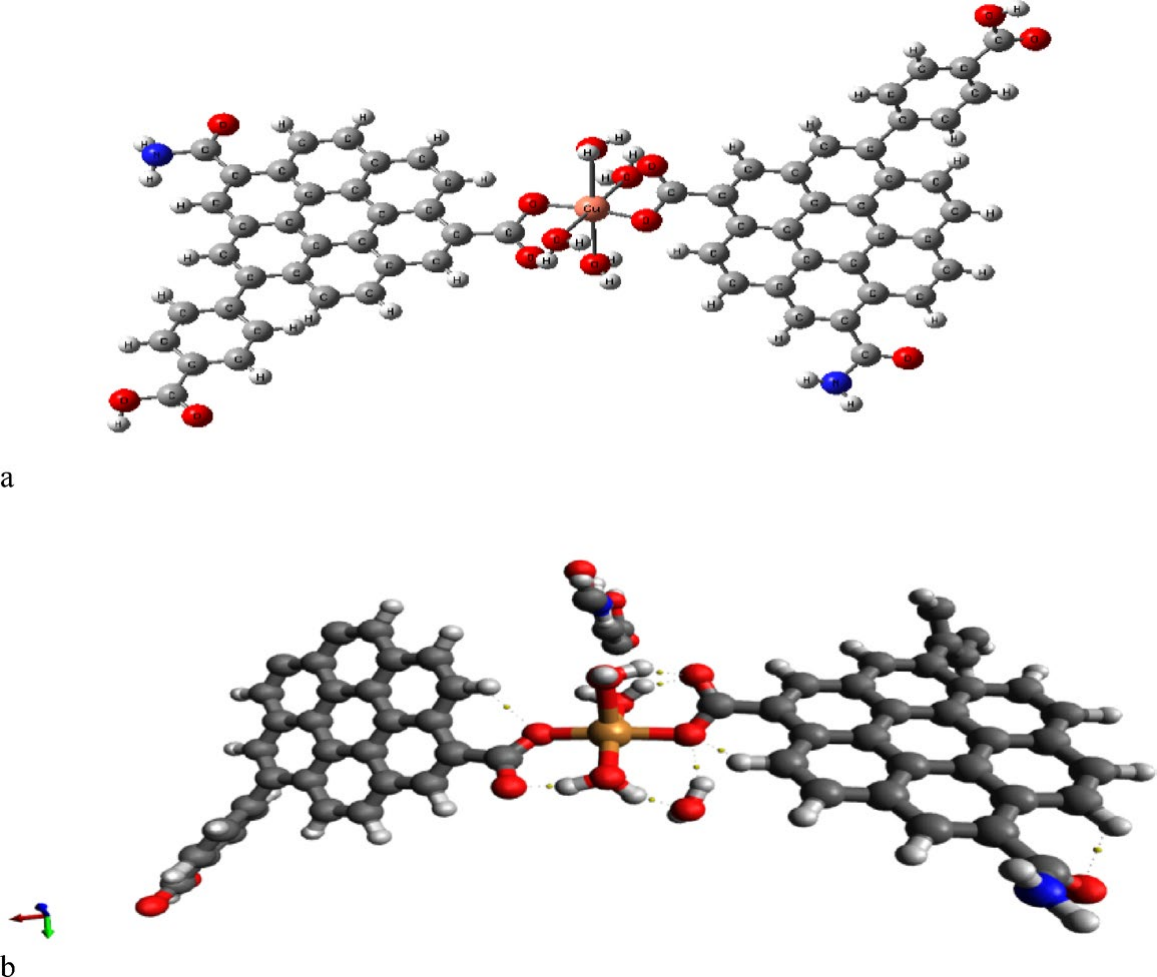
Molecular model for divalent metals which coordinated with humic acid hydrated with 4 water molecules whereas, (**a**) the scheme for the model, (**b**) the QTAIM map for the model indicated in (**a**).

The PM6 data concerning possible coordination between R-COOH as a model and analyzed metals must be verified and generalized. Thus, a general model illustrating the potential coordination between hydrated metals and HA will be introduced. Figure 7a displayed the generalized model, whereas the HA model shown in Fig. 1 replaces the minimized model R-COOH. This is carried out to validate the data obtained with R-COOH and to ensure that R-COOH genuinely represents HA.

Figure 7b illustrates the Quantum Theory of Atoms in Molecules (QTAIM) for the interaction between two humic acid units and a tetra-hydrated Cu.

The QTAIM analysis of the generalized metal-HA model identified the three primary types of bonding interactions within the complex. The covalent bonds are the strong, fundamental links joining the atoms within the molecules. While the coordination bonds connections form between the central Cu atom and the oxygen atoms of two different components: the carboxylate groups (COO−) from the humic acid (HA) units, and the oxygen atoms from the surrounding water molecules. The hydrogen bonds which represented by the yellow dashed lines, these comprise a network of secondary interactions, including bonds between: the carboxylate oxygen and the hydrogen of the coordinated water molecules surrounding the Cu center. The hydrogen atoms of the HA units and the oxygen of the coordinated water molecules. The hydrogen atoms of the amine group on the right HA unit and an adjacent oxygen atom (likely water or another system component). The hydrogen of the hydroxyl group and the oxygen of a carboxylate group on the left HA unit. Collecting this one can conclude that, the QTAIM analysis verifies the presence of covalent bonds, coordination bonds, and different hydrogen bonding interactions, offering insights into their strengths and features based on electron density topology. The left humic acid unit suggested the potential breakdown of functional groups.

In summary, QTAIM converts the visual representation of Fig. 7b into a rigorous, numerical blueprint of the complex’s stability, elucidating the subtle electronic factors that govern the metal’s coordination and the functional role of the water molecules in the system.

## Conclusion

The current DFT computational study presents a model of humic acid HA as an organic molecule containing functional groups. The model was validated as IR calculated at B3LYP/6-31G(d, P) of HA model demonstrates a spectrum that aligns with the FTIR experimental results reported for HA. Results showed that COOH is the reactive part of HA, which simplifies the model to R-COOH. A comparison of DFT, MP2, and the semi-empirical PM6 showed that PM6 offers satisfactory results for this molecular system while requiring minimal computational time. PM6 is additionally employed to illustrate the coordination of Cd, Cu, and Pb with R-COOH. MESP and HOMO/LUMO findings indicated that the coordinated metals with HA remains active after interaction.

The coordination scheme suggested that each divalent metal interacts with two hydrogen bonds from two COOH groups, with each group belonging to an R-COOH model. Hydration occurred so that each metal is associated with 4 water molecules. Hydrated divalent metals associated with two R-COOH behave like standard divalent metals. This computational method demonstrated the appropriateness of using the PM6 method in environments specifically for the coordination of metals with humic acid molecules.

Collecting the data one can conclude that, it can be inferred that humic acid (HA) has oxygen-rich functional groups that facilitate hydrogen bonding with the analyzed metals in either hydrated or non-hydrated states. HA is a plentiful and inexpensive molecule that can easily bind divalent metals in aquatic settings.

Typically, each divalent metal like Cu tends to associate with four water molecules, subsequently forming a complex that involves coordination with two hydrogens from the two COOH groups of HA units.

QTAIM analyses suggested that the interaction between humic acids and hydrated Cu can greatly affect their degradation and stability.

The Molecular Electrostatic Potential (MESP) maps and the HOMO–LUMO orbitals are complementary computational tools. When interpreted together, they provide a powerful explanation for the difference in reactivity between aliphatic and aromatic carboxylic acids (R-COOH) in humic acid (HA). The MESP map pinpoints the electrostatic destination of the incoming metal ion, while the HOMO orbital dictates the kinetic ability of the ligand to donate electrons, with the HOMO–LUMO gap quantifying the ease of reaction.

## Data Availability

The data that support the findings of this study are available from the corresponding author upon reasonable request.
